# Topographical regulation of primary cilia orientation and length in mesenchymal stem cells

**DOI:** 10.1186/2046-2530-1-S1-P34

**Published:** 2012-11-16

**Authors:** R McMurray, MM Knight

**Affiliations:** 1 Queen Mary, University of London, UK

## Introduction

The role of the primary cilia in directing the differentiation of mesenchymal stem cells (MSCs) has recently been demonstrated in response to chemical cues. In addition there is increasing evidence for the role of the primary cilia in sensing environmental cues. The purpose of this study was to test the hypothesis that surface topography, which regulates MSC differentiation, influences primary cilia structure and function.

## Methods

Grooved topographical surfaces were produced by hot embossing a quartz stamp into the polymer polycaprolactone (PCL) and then coating in fibronectin (10ug/ml) to promote even cell attachment. MSCs were cultured for 1 and 3 days in basal media (α-MEM + 10% FBS) followed by 24 hours culture in serum free media on either grooved (540nm deep) or planar surfaces. Cells were fixed and stained with acetylated α-tubulin to label the primary cilia which were visualised using confocal microscopy.

## Results

MSCs and their primary cilia were observed to orientate parallel to the grooves. In addition primary cilia length was found to be significantly different (p<0.005) between the two surfaces with mean lengths 3.1µm and 2.6µm for cells on grooved and planar surfaces respectively.

## Discussion

The mechanism for cilia alignment in response to topography is unclear but is unlikely to be mediated by alignment of the extracellular matrix as suggested in tissue. These changes in primary cilia structure may be involved in mediating stem cell differentiation and other changes in cell function associated with the topographical cues.

